# Tumor Lysis Syndrome in the Chronic Phase of Chronic Myeloid Leukemia Following COVID-19 Infection: A Case Report

**DOI:** 10.7759/cureus.24386

**Published:** 2022-04-22

**Authors:** Adnan Humam Waseem Hajjar, Shahem Abbarh, Abdulrahman Al-Mashdali, Awni Alshurafa, Mohammad Abu-Tineh, Hana Qasim, Khalid Ahmed, Mohamed A Yassin

**Affiliations:** 1 Internal Medicine Department, Hamad Medical Corporation, Doha, QAT; 2 Internal Medicine Department, Hamad Medical Corporation, Riyadh, SAU; 3 Department of Hematology and Medical Oncology, Hamad Medical Corporation, Doha, QAT

**Keywords:** acute kidney injury, rasburicase, cairo and bishop classification, tumor-lysis syndrome, tumor-lysis syndrome in covid-19 infection, tumor-lysis syndrome in chronic myeloid leukemia

## Abstract

Tumor lysis syndrome (TLS) is a hematological emergency. This syndrome is characterized by metabolic derangements such as hyperkalemia and hypocalcemia, which result from rapid lysis of cells, especially rapidly growing tumors, after the initiation of chemotherapy. It is rarely seen in chronic myeloid leukemia (CML) and has not been previously reported to be triggered by coronavirus disease 2019 (COVID-19) infection. We report a case of a 45-year-old male, a known case of CML in the chronic phase, who presented with fatigue and abdominal pain for four days. Initial laboratory results were consistent with leukocytosis and positive COVID-19 antigen. The patient was started on intravenous fluids and hydroxyurea; however, over the next few days, he deteriorated quickly and developed oliguric acute kidney injury (AKI) with electrolyte disturbance consistent with TLS. The patient was shifted to the intensive care unit and underwent one sustained low-efficiency dialysis (SLED) session and received rasburicase. Over the next few days, the patient started to improve and was discharged in good shape. Although CML rarely presents with TLS, physicians should monitor their patients closely, especially those who have concurrent COVID-19 infection, as this condition may result in lethal sequelae such as AKI, severe arrhythmias, and multiorgan failure. Additionally, early detection and treatment lead to a better prognosis.

## Introduction

Tumor lysis syndrome (TLS) is considered a hematological emergency. TLS results from the rapid destruction of tumor cells spontaneously or, more commonly, following the initiation of chemotherapy, causing a massive release of the intracellular contents into the blood. TLS metabolic derangements include hyperuricemia, hyperkalemia, hyperphosphatemia, uremia, and hypocalcemia [1,2]. Usually, these abnormalities occur rapidly, exceeding the body homeostasis, and may result in acute kidney injury (AKI), severe arrhythmias, multiorgan failure, or even death [3].

TLS has been commonly associated with hematological malignancies [4]. In 2010, a risk stratification system for TLS was proposed based on the type of malignancy, the burden of disease, treatment, expected response to treatment, and renal function [5]. High proliferative hematological malignancies such as non-Hodgkin lymphoma and acute leukemias are classified as high-risk diseases in this risk stratification system. In contrast, chronic myeloid leukemia (CML) is considered a low-risk disease for TLS, with only a few case reports published in the literature [6-10].

Since its emergence in 2019, coronavirus disease 2019 (COVID-19) infection has been linked to several hematological problems, including lymphopenia, hypercoagulability state, and disseminated intravascular coagulation [11]. COVID-19 has also been associated with many other diseases such as diabetes mellitus, hypertension, and venous thromboembolism (VTE) [12,13]. However, in our literature review, we did not find any case reports describing TLS triggered by a COVID-19 infection in CML. In this report, we present a case of TLS in a patient with CML following COVID-19 infection.

## Case presentation

A 45-year-old male who was known to have CML in the chronic phase for five years, on imatinib with poor compliance to his medication, presented with a seven-day history of non-specific abdominal pain and fatigue. This was associated with nausea and several episodes of vomiting during the last four days prior to the presentation. His medical history was also significant for hypothyroidism, for which he had been taking levothyroxine 125 mcg. Overall, he was not compliant with his medications. On examination, the patient was vitally stable but looked ill and dehydrated. There was a generalized abdominal tenderness along with hepatosplenomegaly on abdominal examination.

Initial laboratory tests (Table 1) revealed leukocytosis with normal creatinine (Cr) and thyroid-stimulating hormone (TSH). Peripheral blood smear showed 1% blasts. The patient was also found to have a positive COVID-19 antigen. Chest X-ray was unremarkable. He was hydrated with intravenous (IV) normal saline and started on allopurinol 300 mg twice daily and hydroxyurea 1000 mg three times daily. On the next day following the admission, the patient became drowsy; his labs (Table 1) showed an acute drop in WBC count, and his laboratory picture was consistent with TLS (high blood urea nitrogen, Cr, and phosphate, along with low calcium).

**Table 1:** Progression of patient’s laboratory test derangements WBC: white blood cell; BUN: blood urea nitrogen

Lab variables	On admission (day 1)	Day 2	Day 4	Day 6	On discharge (day 11)	Reference range
WBC (x 10^3^/uL)	265	137	137	124	76	4.5–11
BUN (mmol/L)	6.2	16.2	27.2	62	14	2.5–7.8
Creatinine (umol/L)	73	149	152	282	98	62–106
Potassium (mmol/L)	3.5	4.2	4.2	4.5	3.6	3.5–5.3
Phosphorus (mmol/L)	1.65	1.93	2.17	2.81	1.46	0.8–1.5
Calcium (mmol/L)	2.06	1.89	1.97	1.37	2.18	2.20–2.60
Uric acid (umol/L)	346	517	660	70	277	200–430

**Figure 1:**
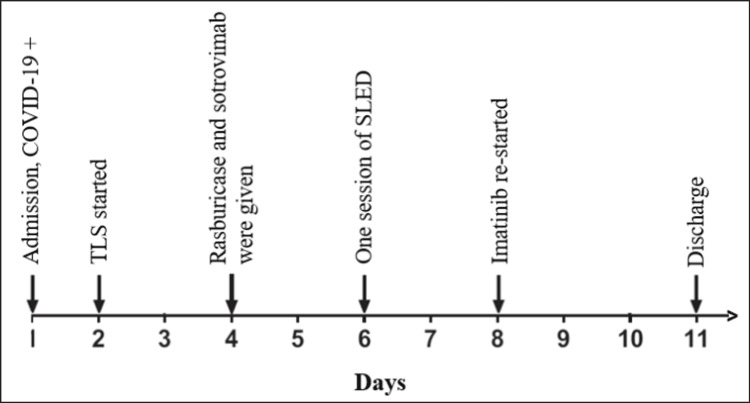
Timeline of important clinical events during the hospital stay COVID-19: coronavirus disease 2019; TLS: tumor lysis syndrome; SLED: sustained low-efficiency dialysis

Despite receiving treatment with IV fluids and allopurinol, the clinical status of our patient deteriorated rapidly, and he was subsequently shifted to the medical ICU. He received one dose of IV rasburicase 10 mg, after which his uric acid returned to the normal level. In addition, he was given one dose of sotrovimab as a treatment for his COVID-19 infection. On day six of admission, the patient underwent one session of sustained low-efficiency dialysis (SLED) due to worsening urine output and AKI. After that, the patient started to improve clinically and lab-wise. Imatinib was re-started with a WBC count of 50 x 10^3^/uL. The patient was discharged in good shape with a significant improvement in his condition. A timeline of the clinical events during his hospital stay is depicted in Figure 1.

## Discussion

CML is a hematological malignancy characterized by dysregulated and increased production rate of mature granulocytes. The hallmark of CML genetic mutation is the abnormal chromosome 22 called the Philadelphia (Ph) chromosome, which results from the fusion of BCR (on chromosome 22) and ABL1 (on chromosome 9) [14]. The BCR-ABL1 fusion gene leads to increased and dysregulated tyrosine activity, the primary mechanism behind cellular proliferation. Based on the pathogenesis of CML, the mainstay of treatment is tyrosine kinase inhibitors such as imatinib, which prevent the progression of the disease [15,16].

TLS is an oncologic emergency that results from a sudden release of intracellular components to extracellular fluid following the lysis of tumor cells. TLS is commonly defined and classified based on Cairo and Bishop criteria, proposed in 2004, and can be classified into laboratory or clinical TLS [2]. Cairo-Bishop's laboratory TLS criteria include two or more of the following features, occurring up to three days before or seven days after chemotherapy: uric acid ≥476 mmol/L, phosphorus ≥1.45 mmol/L, potassium ≥6.0 mmol/L (or a 25% increase from baseline in all), and calcium ≤1.75 mmol/L (or a 25% decrease from baseline). The clinical criteria include one or more of the following features: Cr of more than 1.5 times the upper limit of normal, cardiac arrhythmias, neuromuscular irritability, seizures, or sudden death, which cannot be explained by the direct toxicity of a therapeutic agent. Our patient met both the laboratory and clinical criteria.

TLS is mainly seen in hematological malignancies characterized by significant tumor burden with high proliferative rate, such as acute leukemia and non-Hodgkin lymphoma. TLS can also be observed less frequently in chronic leukemia and solid tumors [17]. In particular, CML is considered a low-risk disease for TLS (<1% risk) based on a risk stratification system by Cairo et al. [5], with TLS incidence in CML patients in the literature confined to a handful of case reports. Generally, TLS is triggered by the initiation of chemotherapy agents or radiotherapy; however, spontaneous TLS without the initiation of any cytotoxic modalities has also been described and may result in more severe clinical outcomes due to the lack of benefit of pretreatment [4]. There are no reported cases of hydroxyurea triggering TLS by itself; however, in our literature review, hydroxyurea has been found to cause TLS along with the administration of either imatinib [10], nilotinib [7,9], or splenic irradiation [6]. Additionally, the effect of cytoreduction therapy in chronic leukemia such as CML is observed after three to seven days and it is less prominent when compared to acute leukemias. Our patient was on long-term imatinib, though he was not compliant; he received only one dose of 1000 mg hydroxyurea before the emergence of the TLS picture.

Interestingly, our patient was found to have a COVID-19 infection upon admission (one day prior to TLS). Although COVID-19 infection has been associated with different hematologic complications in the literature, it was not directly linked to TLS. Given the absence of apparent risk factors for TLS in our patient, the COVID-19 infection was probably the predisposing factor in this case. Although the mechanism of action for COVID-19-induced TLS is unknown, it could be attributed to the overall pro-inflammatory environment created by the infection, which can stimulate tumor cells turnover.

The management of TLS starts with implementing appropriate prophylactic measurements for those at risk of developing TLS. The recommended prophylactic therapy for low-risk diseases such as CML includes monitoring and hydration. Allopurinol, a competitive inhibitor of xanthine oxidase, can be added [5]. Although allopurinol effectively inhibits new uric acid formation, it is ineffective in reducing existing uric acid. In addition, allopurinol is relatively slow to reduce uric acid levels compared to rasburicase. A multicenter study on adults at risk for TLS demonstrated that allopurinol had a significantly lower response rate for uric acid than rasburicase (66% versus 87%). The mean uric acid reduction within four hours was also lower with allopurinol (14%) than rasburicase (88%) [18]. In cases where allopurinol is ineffective for hyperuricemia treatment, rasburicase can be given instead. Rasburicase is a recombinant form of uric oxidase enzyme that breaks down uric acid into allantoin, a significantly more water-soluble substrate. It has been proven to be safe and effective in decreasing uric acid levels in TLS cases [19]. Rasburicase, however, is costly and contraindicated in patients with glucose-6-phosphate dehydrogenase (G6PD) deficiency due to the risk of hemolysis.

Our patient was initially started on IV normal saline and allopurinol as prophylaxis, given the severe leukocytosis and dehydration. Nevertheless, he developed TLS and required rasburicase, after which the serum uric acid level decreased dramatically. Furthermore, hemodialysis might be occasionally needed to manage excessive electrolytes derangement, hyperuricemia, or AKI associated with TLS not responsive to pharmacologic agents [17]. Severe AKI requiring dialysis is estimated to affect 12.8% of patients with TLS based on a nationwide retrospective study that reviewed more than 22,000 patients with TLS in the United States [20]. Our patient underwent one SLED session due to symptomatic AKI and anuria, after which he improved clinically and biochemically.

## Conclusions

This case report discussed a presentation of TLS in CML, which was triggered by COVID-19 infection; a case of this nature has not been previously reported in the literature. This case demonstrates that COVID-19 infection might trigger TLS in CML patients, a life-threatening complication. Patients admitted with hematological malignancies and COVID-19 infection should be closely monitored for electrolytes disturbance as early detection and treatment of TLS lead to a better prognosis.
